# 罗普司亭N01治疗难治性再生障碍性贫血的有效性及安全性：单中心回顾性研究

**DOI:** 10.3760/cma.j.cn121090-20250710-00325

**Published:** 2026-02

**Authors:** 起林 庄, 紫薇 刘, 辰 杨, 苗 陈, 冰 韩

**Affiliations:** 中国医学科学院、北京协和医学院北京协和医院血液科，北京 100730 Department of Hematology, Peking Union Medical College Hospital, Chinese Academy of Medical Sciences & Peking Union Medical College, Beijing 100730, China

## Abstract

罗普司亭N01（QL0911）是一种新型血小板生成素受体激动剂（TPO-RA），其在既往多线治疗失败的难治性再生障碍性贫血（AA）中的应用资料尚有限。本研究回顾性分析2024年5月至12月在北京协和医院接受罗普司亭N01治疗的32例难治性AA患者的临床结局，这些患者既往均接受环孢素A及至少2种口服TPO-RA足量、足疗程治疗无效，且本次罗普司亭N01治疗时间≥3个月、随访时间≥6个月。入组患者中位年龄62（24～79）岁，男性11例（34.4％），30例为输血依赖性非重型AA，2例为重型AA；罗普司亭N01中位治疗时间为5（3～10）个月，中位随访时间为6（6～10）个月。治疗3个月、6个月及末次随访时的总反应率分别为71.9％、75.0％和75.0％，完全反应率分别为28.1％、28.1％和31.2％；达到血液学反应和完全缓解的中位时间分别为1（1～4）个月和3（1～4）个月，提示在难治人群中仍具有较快起效特点。随访期间共有14例（43.8％）发生不良反应，均为1级，经对症处理后好转，未导致减量或停药，亦未见治疗相关肝功能损害、血栓事件或死亡。曾获得疗效的患者中有3例在随访期内复发，2例新发骨髓增生异常综合征相关基因突变，未观察到阵发性睡眠性血红蛋白尿克隆新发或扩增。上述结果表明，即使在既往接受足量环孢素A及多种口服TPO-RA治疗均无效的难治性AA患者中，罗普司亭N01仍可获得较高的血液学反应率且起效迅速，安全性良好，短期内未见明显克隆演化信号。

## Abstract (en)

Romiplostim N01（QL0911）is a novel thrombopoietin receptor agonist（TPO-RA）; however, clinical data regarding its efficacy and safety in patients with refractory aplastic anemia（AA）who have failed multiple prior lines of therapy are limited. This retrospective single center study analyzed the clinical outcomes of 32 patients with refractory AA who received romiplostim N01 at Peking Union Medical College Hospital between May 2024 and December 2024. All patients had previously failed to respond to full dose cyclosporine and at least two oral TPO-RA administered for adequate treatment durations and had been treated with romiplostim N01 for ≥3 months and followed for ≥6 months. The median age of the cohort was 62 years（range, 24–79 years）, and 11 patients（34.4％）were male. Thirty patients had transfusion-dependent non-severe AA（TD-NSAA）, and 2 had severe AA（SAA）. The median duration of romiplostim N01 treatment was 5 months（range, 3–10 months）, and the median follow-up time was 6 months（range, 6–10 months）. The overall response rates（ORR）at 3 months, 6 months, and the last follow-up were 71.9％, 75.0％, and 75.0％, respectively, and the complete response rates（CRR）were 28.1％, 28.1％, and 31.2％, respectively. The median time to hematologic response was 1 month（range, 1–4 months）, and the median time to complete response was 3 months（range, 1–4 months）, indicating a rapid onset of action even in this refractory population. A total of 14 patients（43.8％）experienced adverse events, all of which were grade 1 and improved with symptomatic management; no dose reductions or treatment discontinuations were required, and no treatment related liver dysfunction, thrombotic events, or deaths were observed. During follow-up, relapse occurred in 3 patients who had previously responded（3/26, 11.5％）, and newly detected myelodysplastic syndrome（MDS）-related gene mutations were identified in 2 patients（6.3％）. No new or expanding paroxysmal nocturnal hemoglobinuria（PNH）clones were detected. These findings suggest that romiplostim N01 can induce rapid and high hematologic response rates with a favorable safety profile in patients with refractory AA who have failed full-dose cyclosporine A and multiple oral TPO-RA; no obvious short-term signals of clonal evolution were observed.

再生障碍性贫血（AA）是一种骨髓造血衰竭性疾病，按严重程度分为重型AA（SAA）与非重型AA（NSAA），SAA的一线治疗方法之一为免疫抑制剂（IST），6个月的有效率达60％～80％[1]–[3]，联合血小板生成素受体激动剂（TPO-RA）疗效还可以进一步提升[4]–[5]。然而，仍有部分难治性AA患者在使用足量、足疗程的IST治疗后，甚至联合艾曲泊帕或海曲泊帕等TPO-RA治疗后仍无效[6]。

罗普司亭是一种新型肽类TPO-RA类药物，是由4个TPO-R结合结构域与Fc载体段组成的多肽偶联物，通过结合并激活骨髓内巨核细胞与造血干细胞的TPO-R胞外结构域来激活一系列与TPO类似的下游通路，进而可能实现血小板、血红蛋白、白细胞三系的血液学改善[7]–[9]。目前罗普司亭已在原发免疫性血小板减少症（ITP）、AA、肿瘤化疗相关性血小板减少症（CTIT）等多个疾病中有使用的报道[7],[10]–[11]。在AA中，已有多个国家的临床试验证明其临床有效性[12]–[16]，例如在日本的一项长期随访研究中，使用罗普司亭治疗难治性AA，患者PLT、HGB、ANC与网织红细胞计数均有所改善[13]。

我国自主研发的罗普司亭生物仿制药罗普司亭N01（QL0911）治疗ITP患者的疗效已在Ⅲ期临床试验中得到验证[17]，其疗效与原研的罗普司亭相仿，并在2024年4月获批ITP适应证上市。目前，已有罗普司亭N01治疗CTIT的临床试验报道[18]，但在AA患者中，无论是初治还是难治者，均未见报道。很多难治性AA患者在使用罗普司亭N01之前已使用过足量的其他口服TPO-RA，依然无效。本文回顾性分析了罗普司亭N01在接受足量环孢素A及至少2种口服TPO-RA后，依然无效的难治性AA患者中的疗效与安全性。

## 病例与方法

一、病例资料

本研究为单中心、单臂、回顾性观察性研究。连续收集2024年5月至2024年12月北京协和医院就诊的难治性AA患者，这些患者不具备行异基因造血干细胞移植（allo-HSCT）或抗胸腺细胞球蛋白（ATG）治疗的条件，在使用罗普司亭N01前，至少经过6个月环孢素A 3～5 mg·kg^−1^·d^−1^，及2种足量、足疗程（至少3个月）口服TPO-RA治疗无效。AA诊断及分类符合文献[1],[19]标准，血小板输注、血红蛋白输注依据为：PLT<20×10^9^/L或存在明显出血倾向；HGB<60 g/L或<80 g/L但合并严重心、肺疾病或其他需要输血的疾病。

符合以下标准的患者纳入最终分析：年龄满18周岁，经各种完善检查符合难治性AA［特别除外遗传性AA，低增生骨髓增生异常综合征（h-MDS），有≥50％的阵发性睡眠性血红蛋白尿（PNH）克隆（外周血粒细胞Flaer阴性细胞计数）］；罗普司亭N01用药前至少满足以下3种条件中的1项：HGB<90 g/L、PLT<30×10^9^/L、ANC<0.5×10^9^/L；使用罗普司亭N01前，无活动性感染；如果无反应，需使用足量（每周20 µg/kg）罗普司亭N01至少3个月；使用后需随访至少6个月；除输血及G-CSF（ANC<0.5×10^9^/L使用，>1.0×10^9^/L停用）外，罗普司亭N01是唯一治疗AA的药物；有完整的临床数据、愿意提供并签署数据提供知情同意书者。

二、治疗方案及信息收集

罗普司亭N01起始剂量为每周20 µg/kg，如果未达到部分缓解（PR）以上疗效，需使用至少3个月，达到最佳疗效后，可逐渐减量至停药，或以最低剂量维持，随访时间大于6个月。

收集患者的基线数据，包括人口学资料、病史、体格检查、血常规、生化、骨髓穿刺、骨髓活检、染色体、髓系肿瘤相关基因、PNH克隆、免疫指标、病毒学指标等，并在罗普司亭N01治疗前，治疗后1个月、3个月、6个月以及随访期末收集患者的临床信息（包括药物相关不良反应）、血常规、生化等资料，并每6个月或必要时收集骨髓、活检、染色体及髓系肿瘤相关基因信息。

根据常见不良事件评价标准（Common Terminology Criteria for Adverse Events，CTCAE）5.0评价不良反应。

三、疗效评价

疗效标准参考英国血液学标准委员会制订[1]。复发：达到疗效反应后，出现任一系血液学指标的显著下降或进行性下降，失去治疗反应并需要恢复血小板或红细胞输注或重新恢复初始剂量治疗。

四、随访

随访信息来自住院/门诊病历和电话随访记录。随访起点为患者接受首次罗普司亭N01治疗之日，随访截止时间为2025年6月30日。主要随访指标包括血常规、肝肾功能、以及不良反应与克隆演化情况。

五、统计学处理

本研究仅进行统计学描述，计量资料采用中位数（范围）表示，计数资料采用例数（构成比）表示；使用R 4.4.1，通过ggplot2（v3.5.1）及VennDiagram（v1.7.3）包绘图。

## 结果

一、基线特征

本研究共入组32例患者，年龄62（24～79）岁，男性占34.4％（11/32），30例为输血依赖性（TD）-NSAA，2例为SAA，AA确诊至罗普司亭N01治疗的中位时间为66（6～246）个月。罗普司亭N01治疗前，患者的治疗包括：环孢素A（32例，100％），中位用药时间12（6～132）个月；他克莫司（14例，43.8％），中位用药时间6（4～40）个月；重组人血小板生成素（rhTPO）（21例，65.6％），中位用药时间2（1～3）个月；雄激素（20例，62.5％），中位用药时间25（10～132）个月；艾曲泊帕（13例，40.6％），中位用药时间10（3～17）个月；海曲泊帕（27例，84.4％），中位用药时间3（3～10）个月；阿伐曲泊帕（25例，78.1％），中位用药时间4（3～15）个月；芦曲泊帕（6例，18.8％），中位用药时间4（3～8）个月。

患者基线中位HGB为92（40～104）g/L，中位WBC为3.22（1.01～9.56）×10^9^/L，中位ANC为1.21（0.33～7.23）×10^9^/L，中位PLT为12（2～38）×10^9^/L。基线有3例（9.4％）Flaer阴性中性粒细胞比例大于1％，分别为1.8％、7％、12％。1例（3.1％）患者存在del（15q）核型异常；5例（15.6％）患者存在髓系肿瘤相关基因突变，其中2例为TET2突变，1例为ASXL1突变，1例为ASXL1、TP53突变［单打击突变，等位基因突变频率（VAF）13％］，1例为DNMT3A突变。

二、疗效反应

中位随访6（6～10）个月。罗普司亭N01的中位治疗时间5（3～10）个月，其中62.5％（20/32）患者罗普司亭N01治疗满6个月。整体来看，大部分患者在治疗早期即获得血液学反应。3个月、6个月及末次随访时的总有效率（ORR）分别为71.9％（23/32）、75.0％（24/32）、75.0％（24/32）；完全缓解（CR）率分别为28.1％（9/32）、28.1％（9/32）、31.2％（10/32）。达到OR的中位时间为1（1～4）个月，达到CR的中位时间为3（1～4）个月。

治疗3个月时，13例（40.6％）出现了单系血液学反应，6例（18.8％）出现了两系血液学反应，4例（12.5％）出现三系血液学反应。治疗6个月时，15例（46.9％）出现了单系血液学反应，6例（18.8％）出现了两系血液学反应，3例（9.4％）出现三系血液学反应。至最后一次随访时，11例（34.4％）出现了单系血液学反应，8例（25.0％）出现了两系血液学反应，5例（15.6％）出现三系血液学反应（图1）。

**图1:**
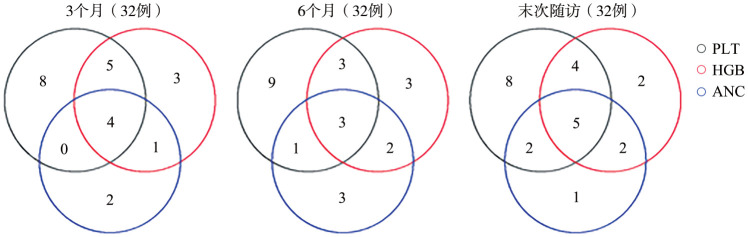
难治性再生障碍性贫血患者罗普司亭N01用药3个月、6个月与末次随访的三系反应维恩图

三、不良反应

至随访期末，共14例（43.8％）患者发生不良反应，分别为上呼吸道感染3例（9.4％）、头痛3例（9.4％）、疲劳3例（9.4％）、注射部位疼痛3例（9.4％）、关节痛2例（6.3％）、肌酐轻度升高1例（3.1％），程度均为1级。对症治疗后好转。

在随访期内，未观察到肝功能受损或凝血功能异常。末次随访的谷氨酸转氨酶中位浓度为14（5～41）U/L，相较基线水平13（5～142）U/L无显著升高。至随访期末，无因不良反应导致减量或停药。

四、复发与克隆演化

在随访时间内，曾获得疗效者中3例（3/26，11.5％）复发，1例患者用药6个月，在减量时复发，提升至原用药量3个月后仍未重新获得血小板反应，PLT继续下降至<20×10^9^/L；1例患者用药5个月，在维持用药时复发，继续维持用药1个月后未见好转，PLT、HGB和WBC均继续下降；1例患者在用药3个月达到CR后停药，于停药2个月后复发，PLT下降至<20×10^9^/L。

随访期内，共2例（2/32，6.3％）新发MDS相关基因突变：1例用药3个月无效，新检测出DNMT3A基因突变（VAF 10.5％），后停药；1例用药6个月后减量时复发，提升至原剂量用药3个月后无效停药，骨髓原始粒细胞比例2％，同时，新检测出RUNX1（VAF 21％）、TET2（VAF 15％）、TP53（VAF 30％）基因突变。无新检出PNH克隆或原有PNH克隆扩增者。随访期间内无患者死亡。

## 讨论

罗普司亭N01（QL0911）作为罗普司亭生物仿制药，其结构、生物活性与药代动力学等特征与罗普司亭高度一致，主要获批用于成人慢性ITP的治疗。不同于其他四种结合于TPO受体跨膜结构域的TPO-RA类小分子药物（艾曲泊帕、阿伐曲泊帕、海曲泊帕与芦曲泊帕）[4]–[5],[20]–[23]，罗普司亭N01是结合于TPO受体胞外结构域的多肽类TPO-RA药物[9],[12]。作用机制上的不同，使罗普司亭N01具有治疗其他TPO-RA无效的难治性AA的潜在可能。本研究结果显示，在经过足量环孢素A及至少2种口服TPO-RA类药物治疗无效的患者中，罗普司亭N01仍具有良好的有效性与安全性。

在本研究中，用药3个月、6个月及随访期末，罗普司亭N01总反应率分别为71.9％、75.0％和75.0％，CR率分别为28.1％、28.1％和31.2％，而原研罗普司亭治疗艾曲泊帕无效的难治性AA研究中在3个月时ORR为76％，中位随访12个月时ORR达70％，CR率达10％[23]–[24]。本团队的一项研究表明在同样经环孢素A及其他2种TPO-RA治疗无效后，使用原研罗普司亭可在3个月后达到72.7％的ORR与45.5％的CR率[25]。上述结果与本研究结果一致。而使用阿伐曲泊帕治疗艾曲泊帕难治性AA患者在用药3个月与6个月后ORR仅为50％～70％[26]–[27]。相较之下，提示罗普司亭N01在环孢素A及多种口服TPO-RA无效的患者群体中，仍具有较为可观的临床获益。

与其他TPO-RA类药物相似，罗普司亭N01可有除血小板外的其他血细胞系的反应，有些甚至有三系反应。本研究中3个月、6个月及随访期末达到三系反应的患者分别占12.5％、9.4％与15.6％，略低于日本原研罗普司亭治疗艾曲泊帕难治性AA研究中的三系反应率（3个月及随访期末分别为19.0％和23.8％）以及本团队原研罗普司亭研究中的三系反应率（3个月、6个月及末次随访的均达27.3％）[23],[25]。这一差异可能与本研究中患者的难治性背景有关。而使用阿伐曲泊帕治疗难治性AA的研究中用药3个月、6个月及随访期末的三系反应率可达16％、20％与25％[27]，使用海曲泊帕治疗难治性AA的研究中，在18周、24周与52周的三系反应率为10.9％、16.4％、21.8％[28]。对比上述研究，可见罗普司亭N01用于治疗环孢素A及两种口服TPO-RA难治性AA患者具有良好的三系反应率。

罗普司亭N01在随访期间有较好的安全性，共观察到43.8％的不良反应发生率，程度均为1级，经对症治疗后均好转，均未影响罗普司亭N01用药。随访期间未观察到治疗相关的严重不良反应发生，也未观察到治疗相关的肝功能损害或血栓形成事件，与原研罗普司亭的结果一致[12]–[14],[25]。

本研究有3例（11.5％）患者复发。这与其他原研的罗普司亭数据类似，如本团队使用罗普司亭治疗难治性AA的随访研究中，有1例（1/8）于随访5个月后因停药而复发[25]。由于随访时间不同，可能存在复发率的差异。

在本研究中有2例（2/32）新发MDS相关基因突变或染色体异常，而日本的罗普司亭治疗难治性AA随访研究中，在用药27周后共检出2例染色体异常（2/31），与本研究的结果相似[12]。本研究中无新检出PNH克隆或原有PNH克隆显著扩增，与原研罗普司亭治疗难治性AA的结果一致[15],[25]。

本研究存在一定局限性。首先，本研究为回顾性研究，样本量小，存在患者选择偏倚；随访时间较短，无法准确判断复发及克隆演化情况；本研究的患者大多为TD-NSAA，前期的IST仅为环孢素A，存在IST不充分情况，其结果也无法直接应用于ATG+环孢素A+TPO-RA的群体。尽管如此，本研究初步显示了罗普司亭N01治疗难治性AA的有效性与安全性，为环孢素A及口服TPO-RA类药物难治的AA患者提供了新的选择。
